# Optimization of the SARS-CoV-2 ARTIC Network V4 Primers and Whole Genome Sequencing Protocol

**DOI:** 10.3389/fmed.2022.836728

**Published:** 2022-02-17

**Authors:** Arnold W. Lambisia, Khadija S. Mohammed, Timothy O. Makori, Leonard Ndwiga, Maureen W. Mburu, John M. Morobe, Edidah O. Moraa, Jennifer Musyoki, Nickson Murunga, Jane N. Mwangi, D. James Nokes, Charles N. Agoti, Lynette Isabella Ochola-Oyier, George Githinji

**Affiliations:** ^1^Kenya Medical Research Institute (KEMRI)-Wellcome Trust Research Programme (KWTRP), Kilifi, Kenya; ^2^Department of Biological Sciences, University of Warwick, Coventry, United Kingdom; ^3^Nuffield Department of Medicine, Pwani University, Kilifi, Kenya; ^4^Nuffield Department of Medicine, University of Oxford, Oxford, United Kingdom; ^5^Department of Biochemistry and Biotechnology, Pwani University, Kilifi, Kenya

**Keywords:** ARTIC V4, SARS-CoV-2, whole genome sequencing, amplicon drop-offs, protocol

## Abstract

**Introduction:**

The ARTIC Network's primer set and amplicon-based protocol is one of the most widely used SARS-CoV-2 sequencing protocol. An update to the V3 primer set was released on 18th June 2021 to address amplicon drop-off observed among the Delta variant of concern. Here, we report on an in-house optimization of a modified version of the ARTIC Network V4 protocol that improves SARS-CoV-2 genome recovery in instances where the original V4 pooling strategy was characterized by amplicon drop-offs.

**Methods:**

We utilized a matched set of 43 clinical samples and serially diluted positive controls that were amplified by ARTIC V3, V4 and optimized V4 primers and sequenced using GridION from the Oxford Nanopore Technologies'.

**Results:**

We observed a 0.5% to 46% increase in genome recovery in 67% of the samples when using the original V4 pooling strategy compared to the V3 primers. Amplicon drop-offs at primer positions 23 and 90 were observed for all variants and positive controls. When using the optimized protocol, we observed a 60% improvement in genome recovery across all samples and an increase in the average depth in amplicon 23 and 90. Consequently, ≥95% of the genome was recovered in 72% (*n* = 31) of the samples. However, only 60–70% of the genomes could be recovered in samples that had <28% genome coverage with the ARTIC V3 primers. There was no statistically significant (*p* > 0.05) correlation between Ct value and genome recovery.

**Conclusion:**

Utilizing the ARTIC V4 primers, while increasing the primer concentrations for amplicons with drop-offs or low average read-depth, greatly improves genome recovery of Alpha, Beta, Delta, Eta and non-VOC/non-VOI SARS-CoV-2 variants.

## Introduction

Genomic sequencing of Severe Acute Respiratory Syndrome Coronavirus 2 (SARS-CoV-2) has been instrumental in understanding the biology, emergence and spread of the virus globally (1–3). SARS-CoV-2 genomes help explain virus evolution and transmission (4, 5), identify sites on the genome that may aid vaccine/antibody evasion and inform vaccine design (6), improve design of molecular and serological assays (7) and influence public health policy (8).

There are several approaches used for whole genome sequencing (WGS) of SARS-CoV-2 and can be broadly categorized as targeted and non-targeted i.e., metagenomic approaches (9–12). Early SARS-CoV-2 genomes were generated using a metagenomic approach given the lack of reference genome at the beginning of the pandemic (9). Amplicon based methods using SARS-CoV-2 specific primers that amplify between 400 to 2,500 base pairs were designed and implemented using multiplex RT-PCR methods followed by WGS using platforms such as Oxford Nanopore Technologies and Illumina (11–13).

The most widely adopted targeted amplicon approach for SARS-CoV-2 genomic sequencing is the ARTIC protocol. This protocol was developed based on an earlier strategy for sequencing single-stranded RNA viruses from high cycle threshold (Ct) clinical samples (14). It employed an early draft version of the SARS-CoV-2 genome and incorporated two sets of primer pools for efficient multiplexing (15, 16). The protocol had five key steps; (i) cDNA synthesis using superscript IV kit, (ii) multiplex RT-PCR using Q5 kit and ARTIC V1 primers in two pools, (iii) RT-PCR clean-up using beads, quantification and normalization, (iv) native barcode ligation and (v) sequencing on the MINION device. The first version of this protocol was released to the public on 22nd January 2020 and comprised of what became the ARTIC V1 primer-set that consisted of 98 primer pairs spanning the ~30kb except for the 3' and 5' regions. The ARTIC V1 protocol and primer-set had a number of challenges including drop-offs at amplicons 18 and 76 due to primer dimers (17). Subsequently, an improved set of ARTIC V2 primers were released. The V3 primer were released on 24th March 2020 together with an improved overall sequencing approach (13). The V3 primer set contained additional alternate primers added to the V1 primer sets and provided over 50X coverage in all amplicons compared to V1 and V2 primer-sets (13). The ARTIC V2 protocol (GunIt) was quickly replaced by the V3 protocol (LoCost) which was developed to circumvent the huge cost of sequencing during the pandemic. The reagents' cost of SARS-CoV-2 WGS using Nanopore devices has been estimated to be between $11.50 to $35.88 for one sample when calculated based on 96 samples per sequencing run (18–20).

As of 21st November 2021, there were over 5.3 million SARS-CoV-2 genomes shared on the Global Initiative on Sharing All Influenza Data (GISAID) database (21) but only 76% had high genome coverage (≥99%). In Africa, there were over 60,000 genomes but only 43% of the genomes had high coverage. The ability to generate near-complete genomes when using the ARTIC Network's V3 primers, is affected by sample quality, viral load quantity and consistent virus evolution that guarantees mutations on primer binding sites leading to amplicon drop-offs in up to twelve amplicon primer sites across the Delta, Alpha and Beta variants (22, 23). Attempts to improve genome recovery by using supplemental primers or increasing primer concentrations do not always ensure success and can be a challenge (18, 23). The ARTIC Network's V4 primers were released to address mutations in the primer binding sites that were resulting in amplicon drop-offs in the Delta variant of concern (VOC) (24).

The ARTIC V4 primers have shown considerable improvement in the genome recovery of the Delta VOC except at amplicon 90 (23). Here, we report on our in-house optimization of a modified version of the ARTIC Network V4 primers herein referred to as optimized V4, to improve on SARS-CoV-2 genome recovery where the original ARTIC V4 pooling strategy did not yield full genomes and was characterized by amplicon drop-offs.

## Materials and Methods

### Ethics Statement

Samples for SARS-CoV-2 whole genome sequencing study protocol were reviewed and approved by the Scientific and Ethics Review Committee (SERU) residing at the Kenya Medical Research Institute (KEMRI) headquarters in Nairobi (SERU #4035).

### Sample Selection

A total of 43 SARS-CoV-2 positive samples (collected as a combined nasopharyngeal and oropharyngeal (NP/OP) swab) previously sequenced using the ARTIC Network nCoV-2019 V3 primers (24) were selected. These samples had a real-time RT-PCR cycle threshold (Ct) value between 12.6 and 30.7 (median 21.7) based on the spike (S) gene assay from the commercially available RADI COVID-19 detection kit (KH Medical Co. Ltd, South Korea). The genome sequences recovered from these samples were classified as described in Supplementary Table 1.

### RNA Extraction and cDNA Synthesis

Ribonucleic acid (RNA) was extracted from 140 μl of the NP/OP samples using the QIAamp Viral RNA Mini Kit (QIAGEN, cat 52906, Manchester, United Kingdom) according to the manufacturer's instructions. RNA was isolated from a heat-inactivated, cultured SARS-CoV-2 supernatant donated by Aix-Marseille University (Marseille, France) and its genome classified as lineage B.1, which was used as the positive control. The RNA from the positive control sample was labeled PC neat and used to create two sets of 10-fold dilution series herein referred to as PC 1:10 and PC 1:100.

These three positive controls, the 43 samples and a no reverse-transcriptase control (NRT) were used for cDNA synthesis using 2 μl of LunaScript RT Mix (NEB, E3010, MA, USA) and 8 μl of RNA. This reaction was incubated at 25°C for 2 min, 55°C for 10 min, 95°C for 10 min then held at 4°C.

### Primer Reconstitution and Pooling

The lyophilized 218 V3 and 198 V4 primers (Eurofins Genomics, Germany), were resuspended in nuclease-free water according to the oligonucleotide synthesis reports to achieve a stock concentration of 100 μM. We generated two primer pools by combining 5 μl (1X volume) of each primer, where odd and even region primers constituted Pool A and Pool B, respectively. To solve the amplicon drop-offs and uneven coverage problems when deploying the V3 primers, we created a third pool, herein referred as Pool C. This pool comprised primer pairs from regions 3, 9 alternate, 17, 26, 64, 66, 67, 68, 74, 76, 88, 91, and 92 that were also present in pools A and B.

For the V4 primer scheme, a pooling guide was recommended by the developer to mitigate uneven coverage (24). Following the amplicon drop-offs and low coverage depths (<50) for regions covered by primers 5, 8, 21, 23, 76, and 90 in the V4 primers, we increased the volumes of these primers in the respective pools. The primers were added into the reaction at 5X (25 ul) volume for 8, 17, 21, 23, 27, 30, 61, 74, 76 and 90 regions, and at 2X (10 ul) volume for 5, 13, 45 and 79.

### Multiplex RT-PCR

The resulting primer pools were diluted in nuclease-free water to produce 10 μM stock with each primer being utilized at a final concentration of 0.015 μM for the multiplex RT-PCR. For amplification using the V3 primer pools, there were three reactions per sample that were set up by combining 3μl of nuclease-free water, 6.25 μl of Q5® Hot Start High-Fidelity 2X Master Mix (NEB M0494, MA, USA), 2 μl of primer pool and 1.3 μl of cDNA. The V4 primer pools amplification employed two reactions per samples and the reaction components comprised 3 μl of nuclease-free water, 6.3 μl of Q5® Hot Start High-Fidelity 2X Master Mix (NEB M0494, MA, USA), 1.9 μl of primer pool and 1.3 μl of cDNA. The total reaction volume for the multiplex RT-PCR was carried out at half the recommended amount from the ARTIC LoCost protocol (15) and the thermocycling conditions were as follows: 1 cycle of 98°C for 30 s, followed by 25 cycles of 98°C for 30 s and 65°C for 5 min, 15 cycles of 62.5°C for 5 min and 98°C for 15 s, 1 cycle of 62.5°C for 5 min and held at 4°C indefinitely. In addition to the 43 samples and three positive controls from above, a single no template control (NTC) i.e., mastermix only and a single negative control (water + mastermix) were included to serve as an indicator of extraneous nucleic acid contamination.

RT-PCR products from pools A and B of V4 primers were combined to make up a total of 25 μl and cleaned up using 1X AMPure XP beads (Beckman Coulter, A63881, Indianapolis, USA) as highlighted in the amplicon clean-up protocol (15). Since the V3 primers had an additional pool with fewer primer pairs, only 3 μl of the pool C amplicons were added to the amplicons from pools A and B to make up a volume of 28 μl and cleaned using 1X AMPure XP beads (Beckman Coulter, A63881, Indianapolis, USA). The pellet was resuspended in 20 μl of nuclease-free water, and 1 μl of the eluate was quantified using the Qubit dsDNA HS Assay Kit (ThermoFisher, Q32854, California, USA) as stipulated in the manufacturer's handbook.

To reduce the number of samples with low virus abundance proceeding to library preparation we devised an *ad hoc* quality control strategy based on the concentration of the NTC which is usually primers and artifacts. For example, the criteria for grading the amplicons generated using the modified V4 primer pools, and an NTC with a concentration of 27.2 ng/μl were as follows: grade one, ≥ 62 ng/μl, grade two, 28–62 ng/μl and grade three, <28 ng/μl. Samples that fell within the same grade were assigned to one sequencing run, while excluding all grade 3 samples in downstream processes. However, the negative controls were added to all the runs regardless.

### Library Preparation and Nanopore Sequencing

Normalization was performed by adding 7 μl nuclease-free water to 5 μl of cleaned-up RT-PCR amplicons for grade 1 samples. For grade 2 samples, we used 7 μl of the cleaned-up RT-PCR amplicons and topped up with 5 μl of nuclease-free water, whereas 9 μl of the amplicons was used for the negative controls. The end repair and A-tailing of the amplicons was carried out with the Ultra II End repair/dA-tailing Module (NEB, E7546, MA, USA) reagents. The end-prep reaction for each biological sample comprised 1.5 μl of the reaction buffer, 0.5 μl of the enzyme mix and 12 μl of the normalized RT-PCR products. Thermocycling conditions were set at 20°C for 15 min, 65°C for 15 min and 4°C for 1 min.

Barcode ligation employed 1.25 μl of a unique native barcode from EXP-NBD196 (Oxford Nanopore Technologies, Oxford, UK), 2.75 μl of nuclease-free water, 5 μl of Blunt/TA Ligase Master Mix (NEB, M0367) and 1 μl of end-prepped DNA. This mixture was incubated at 20°C for 20 min, 65°C for 10 min and 4°C for 1 min. All the barcoded samples were pooled together and cleaned using 0.4X AMPure XP beads and 250 μl Short Fragment Buffer (ONT) as described in the LoCost protocol (15). The pellet was resuspended in 34 μl of nuclease-free water (0.07X of the total volume of pooled samples), and 1 μl was used for quantification using the Qubit dsDNA HS Assay Kit.

For adapter ligation, about 50 ng of the pooled barcoded sample was utilized along with the Quick Ligation Module (NEB, E6056, MA, USA) reagents and AMII (ONT, Oxford, UK). The components volumes were halved making up a total reaction of 25 μl. This reaction was incubated at room temperature for 20 min, and then cleaned using 1X AMPure XP beads and 125 μl Short Fragment Buffer as described above. Following elution in 15 μl of Elution Buffer (ONT, Oxford, UK), the library was quantified and normalized to 15 ng. The loading library was prepared by adding 37.6 μl of the Sequencing Buffer (ONT), 25.4 μl of the Loading Beads (LB) and 12μl of the library. A SpotON flow cell (ONT, FLO-MIN106D, Oxford, UK) that had more than 800 pores was primed and loaded with 75 μl of the library. The experiment was set up on a GridION Mk1 Sequencing Device using MinKNOW (version 21.05.20). The run was stopped once we had 100,000 reads per sample.

### Analysis

We adopted the ARTIC bioinformatics protocol using the applicable primer scheme to generate consensus sequences (25). Lineage assignment was done using the command-line-based Pangolin (pangolin version 3.1.16, pangoLEARN version 18/10/2021). NextClade (version 0.13.0) was used for clade assignment and overall quality control metrics are shown in Supplementary Table 1. All statistical analysis was done using R version 4.1.1 (26).

## Results

### Effect of Modifications in ARTIC V3 Primers Pooling Strategy

**Figure 1:**
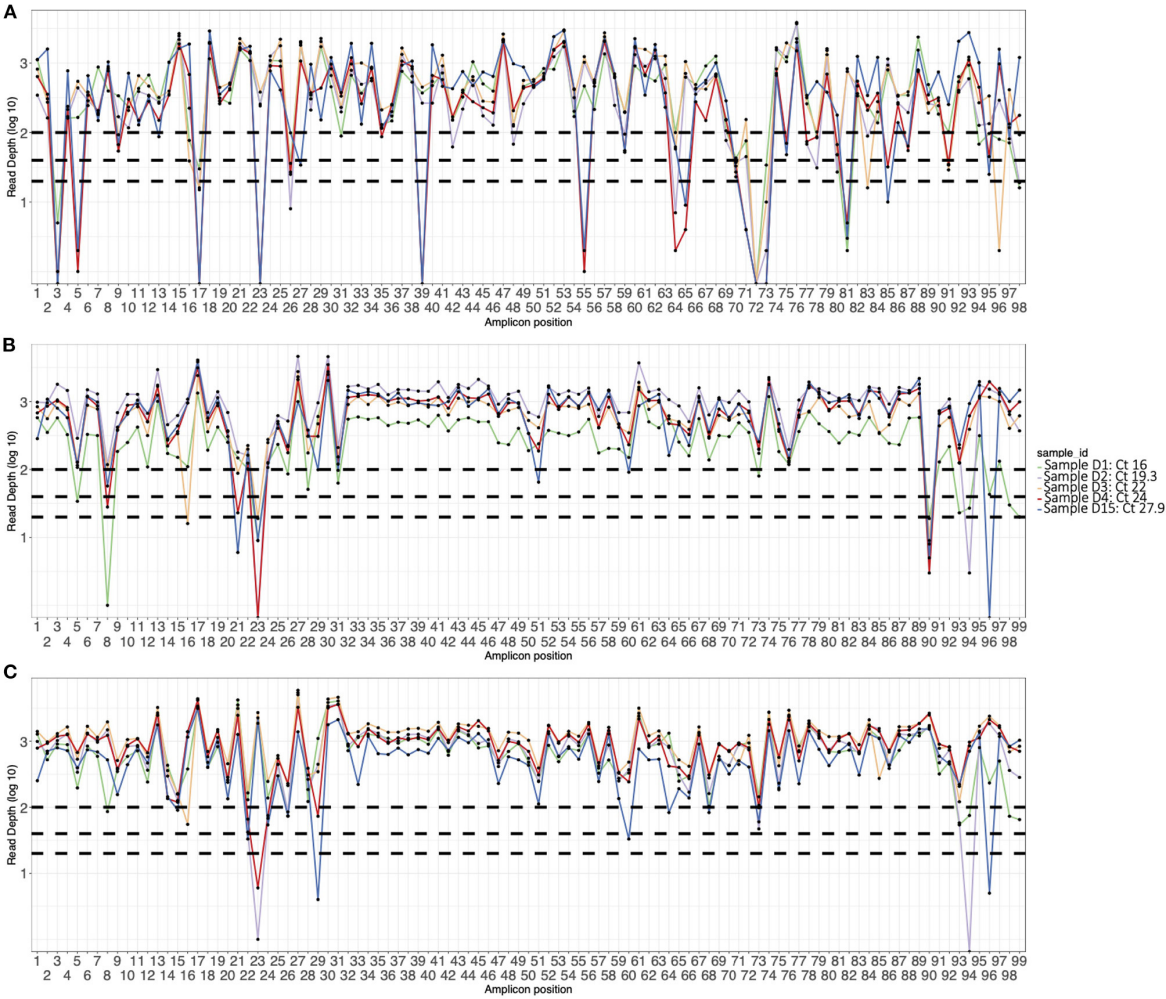
Amplicon plots of five sequences generated using **(A)** V3 primers, **(B)** V4 primers, and **(C)** optimized V4 primers and classified as Delta VOC. The curves show average depth in log scale (y axis) per amplicon (x axis). The horizontal dotted lines indicate amplicon depth cut-offs at 23, 50, and 100.

The initial V3 primers were pooled into two pools (A and B) and had amplicon drop-offs at position 3, 9, 17, 23, 24, 26, 64, 67, 68, 71, 74, 76, 88, 91, and 92 (data not shown). We modified the pooling strategy by creating a third pool as described in the methods. However, drop-offs were observed more frequently in Delta VOC sequences at amplicons 3, 5, 17, 23, 39, 55, 64, 71, 72, 73, 81, and 85 (Figure 1). For the Alpha VOC, amplicon drop-offs were observed at amplicons 3, 17, 23, 64, 70 and 73 (Supplementary Figure 1). For the Beta VOC and Eta variant of interest (VOI), amplicon drop-offs were observed at amplicon 3, 7, 17, 59, and 85 (Supplementary Figure 1).

### Improved Genome Recovery Using ARTIC V4 Primers

**Figure 2:**
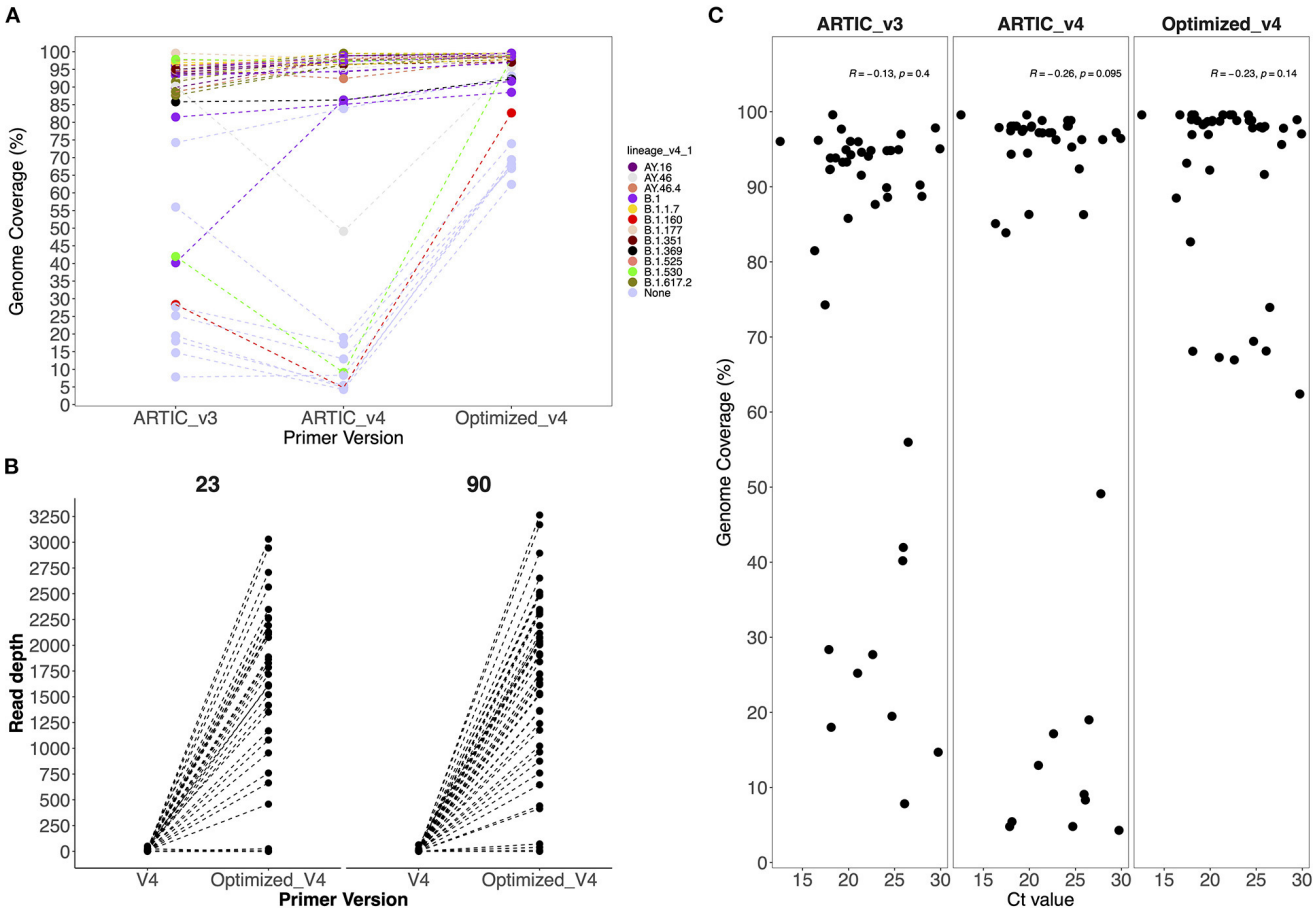
**(A)** Comparison of genome coverage across all sequences generated by the V3, V4 and optimized V4 primers and **(B)** Comparison of the average amplicon depth from amplicons 23 and 90 when using V4 and optimized V4 primers. Primer version is on the x-axis and amplicon depth on the y-axis. **(C)** Comparison of Ct values against genome coverage for V3, V4 and optimized V4 primer sets. Each dot represents a sample whose Ct value is highlighted in the x axis.

No SARS-CoV-2 genomes were recovered from the negative control and non-template control. The ARTIC V4 primers improved genome recovery among the Alpha, Beta, Delta, Eta and non-VOC/VOI variants. We observed a 0.5% to 46% increase in genome recovery in 67% of the samples when using the ARTIC V4 primers compared to the V3 primers (Figure 2A). Amplicon drop-offs at primer positions 23 and 90 were observed consistently for all variants and positive controls (Supplementary Figure 2). Additionally, amplicons 5, 8, 21, 31 and 76 had amplicon depth of <50 in some samples and showed potential of becoming drop-offs especially in samples with Ct values of >25.

### Effect of Increased Primer Volumes in Amplicon Depth and Coverage

To avoid drop-offs in the above stated amplicons, we increased the primer concentrations in the ARTIC V4 set during pooling as described in methods. There was an improvement in 75 and 93% of the genomes after increasing the primer concentrations five times for amplicons 23 and 90, respectively (Figure 2B). However, most of the genomes (>90%) that had no read coverage for amplicons 23 and 90 did not improve despite using the optimized V4 primers.

Generally, there was an improvement of up to 60% in genome recovery across all samples. In 72% of the samples, ≥95% of the genome was recovered. However, only 60–70% of the genomes could be recovered in samples that had performed poorly (<28% genome coverage) with the ARTIC V3 primers (Supplementary Table 2).

### Changes in Lineage Assignment With Increased Genome Completeness

In six sequences, there were changes in lineage assignment with improved genome recovery. Two sequences that were classified as AY.43 and AY.16 lineages were reassigned to B.1.617.2, and one sequence classified as B.1.36.35 was reassigned to B.1. Three sequences that were not assigned a lineage earlier ended up being classified as B.1, B.1.530 and B.1.160 following an increment (>50%) in genome recovery (Table 1).

**Table 1:** A table showing changes in lineage assignment and genome completeness among discrepant samples.

**Sample_ID**	**Lineage with V3**	**Genome coverage with V3 (%)**	**Lineage with optimized V4**	**Genome coverage with optimized V4 (%)**	**Ct value**
sample_d17	AY.16	88.7	B.1.617.2	97.8	27.96
sample_d16	AY.43	91.5	B.1.617.2	99.6	21.38
sample_nv2	B.1.36.35	74.2	B.1	93.1	17.45
sample_nv1	None	28.3	B.1.160	82.7	17.86
sample_nc1	None	41.9	B.1.530	98.0	25.94
sample_nc2	None	40.2	B.1	91.6	25.88

### Comparison of Ct Values and Genome Coverage

There was no observed significant correlation (*p* > 0.05) between the Ct values and genome coverage when using either primer version (Figure 2C). Using a serially diluted PC, genome completeness was low in the PC-neat compared to PC 1:10 and PC 1:100 with amplicon drop-offs observed toward the 3' end of the genome (Supplementary Figure 2).

## Discussion

Amplicon drop-offs that are caused by primer competition have been an issue when sequencing SARS-CoV-2 using the ARTIC tiling primers as previously described (17). With the dominance of the Delta VOC globally, the ARTIC V3 primers had up to 13 amplicon drop-offs when sequencing samples with the Delta VOC in our analysis. The optimized ARTIC V4 primers generated sequences with the highest genome coverage compared to the ARTIC V3 and V4 primers for samples with either VOCs, VOIs or non-VOC/non-VOI. The findings suggest improved genome coverage when using the modified ARTIC V4 primers compared to either ARTIC V3 primers or ARTIC V4 primers. The Omicron variant has up to 10 mutations that may affect the efficiency of the ARTIC V4 primers, but this can be resolved using the V4.1 primers (27).

Increasing the concentrations of primers for regions with low read depth or no amplification improved genome recovery in those regions. We speculate that these primers encountered competition from other primers, hence leading to amplicon drop-offs and increments in primer concentrations improved the read depth at these positions.

Previous studies have reported successful genome recovery in samples with low Ct values (<25) (28, 29). Our findings indicated that there was no significant correlation between genome coverage and Ct value when using either version of the ARTIC primers. Genomes with >95% coverage were recovered from samples with higher Ct values (24–29), and the differences observed could be either due to sample-to-sample variation or batch processing. Therefore, when using the optimized V4 primers, genome completeness (>95%) can be expected for samples with a wide Ct value range (14–29) regardless of the lineage.

Accurate lineage assignment using Pangolin may rely on key single nucleotide polymorphisms in the genome and if these are absent, incorrect lineage assignment is likely to occur (30). Improvements in genome recovery led to the assignation of lineages to three sequences that could not be previously assigned, hence helping identify the variants present in those samples. In three other sequences, the lineages AY.16/AY.43 and B.1.36.35 were reassigned to their parental lineages B.1.617.2 and B.1, respectively. The mutation A28299T is characteristic of lineage AY.43 suggesting that misclassification occurred due to low genome coverage. Additionally, the sequence that was reassigned to B.1.617.2 from AY.16 had a T26076A mutation that is characteristic of AY.16. The parental lineage reassignment suggests that the sequence could either belong to the B.1.617.2 or any other descendant lineage of B.1.617.2 (30). Previously, it has been reported that when using ARTIC V4 primers systematic errors might lead to a T15521A and T8835C mutations (31), but this were not observed in our analysis.

These findings have limitations. First, increasing primer concentrations may lead to a rapid depletion of some primer combinations, raising the overall sample processing cost. Currently, the per-sample cost of our method is estimated to be $18, which is within range of other short-read, amplicon-based approaches for SARS-CoV-2 sequencing. This expense could be reduced if the post RT-PCR clean-up and normalization steps are removed in favor of the RT-PCR dilution step. Secondly, elevated primer concentration by virtue of elevated volumes might have led to over representation of certain fragments over others. However, this can be mitigated by normalizing the fragments across the genome. Moreover, extracting archived samples on different days has an impact on RNA quality, particularly for samples with low viral loads, which impacts downstream sequencing outputs.

In conclusion, implementing the ARTIC V4 and increasing the primer concentrations for amplicons with drop-offs or low average read-depth greatly improved genome recovery among Alpha, Beta, Delta, Eta and non-VOC/non-VOI SARS-CoV-2 variants.

## Data Availability Statement

The datasets presented in this study can be found in online repositories. The names of the repository/repositories and accession number(s) can be found below: 10.7910/DVN/VYPOOP. The accession numbers are: EPI_ISL_4880648, EPI_ISL_7850847, EPI_ISL_7850848, EPI_ISL_7850849, EPI_ISL_7850850, EPI_ISL_7850851, EPI_ISL_7850852, EPI_ISL_7850853, EPI_ISL_7850854, EPI_ISL_7850855, EPI_ISL_7850856, EPI_ISL_7850857, EPI_ISL_7850858, EPI_ISL_7850859, EPI_ISL_7850860, EPI_ISL_7850861, EPI_ISL_7850862, EPI_ISL_7850863, EPI_ISL_5797353, EPI_ISL_5797363, EPI_ISL_5797364, EPI_ISL_5797299, EPI_ISL_5797303, EPI_ISL_5797365, EPI_ISL_5797338, EPI_ISL_5797345, EPI_ISL_5797346, EPI_ISL_5797348, EPI_ISL_4036986, EPI_ISL_4036991, EPI_ISL_4196981, EPI_ISL_4196983, and EPI_ISL_4196993.

## Ethics Statement

The studies involving human participants were reviewed and approved by Scientific and Ethics Review Committee (SERU) residing at the Kenya Medical Research Institute (KEMRI) headquarters in Nairobi (SERU #4035). Written informed consent for participation was not required for this study in accordance with the national legislation and the institutional requirements.

## Author Contributions

GG, CA, LO-O, and DN supervised this study. JM assisted in sourcing the samples. AL, KM, LN, JMM, MM, and TM processed the samples. AL and NM performed data curation. AL and KM analyzed the data and drafted the main manuscript text. All authors contributed toward revising the manuscript. All authors contributed to the article and approved the submitted version.

## Funding

We are grateful for funding support from the National Institute for Health Research (NIHR) (project reference 17/63/82) and 16/136/33 using UK aid from the UK Government to support global health research, the UK Foreign, Commonwealth and Development Office and Wellcome Trust (grant# 102975; 220985) provided reagents to support the sequencing work. Funding for reagents was also provided by the Africa CDC and African Society for Laboratory Medicine (ASLM) and WHO-Afro. GG is funded and supported by NIHR funded GeMVi and TIBA projects (Grant numbers 17/63/82 and 16/136/33). CA is supported through the DELTAS Africa Initiative (DEL-15-003). The DELTAS Africa Initiative is an independent funding scheme of the African Academy of Sciences (AAS), Alliance for Accelerating Excellence in Science in Africa (AESA) and supported by the New Partnership for Africa's Development Planning and Coordinating Agency (NEPAD Agency) with funding from the Wellcome Trust (107769/Z/10/Z) and the UK government.

## Author Disclaimer

Views expressed in this publication are those of the authors and not necessarily those of AAS, NEPAD Agency, Wellcome Trust or the UK government.

## Conflict of Interest

The authors declare that the research was conducted in the absence of any commercial or financial relationships that could be construed as a potential conflict of interest.

## Publisher's Note

All claims expressed in this article are solely those of the authors and do not necessarily represent those of their affiliated organizations, or those of the publisher, the editors and the reviewers. Any product that may be evaluated in this article, or claim that may be made by its manufacturer, is not guaranteed or endorsed by the publisher.
