# Supplementary material for: Optimization of the SARS-CoV-2 ARTIC Network V4 Primers and Whole Genome Sequencing Protocol
Source: Front Med (Lausanne). 2022 Feb 17;9:836728. doi: 10.3389/fmed.2022.836728 (PMC8891481; doi:10.3389/fmed.2022.836728)
Supplement: Supplementary file 1 [file Data_Sheet_1.docx]

Supplementary Material

# Supplementary Figures and Tables.

## Supplementary Figures


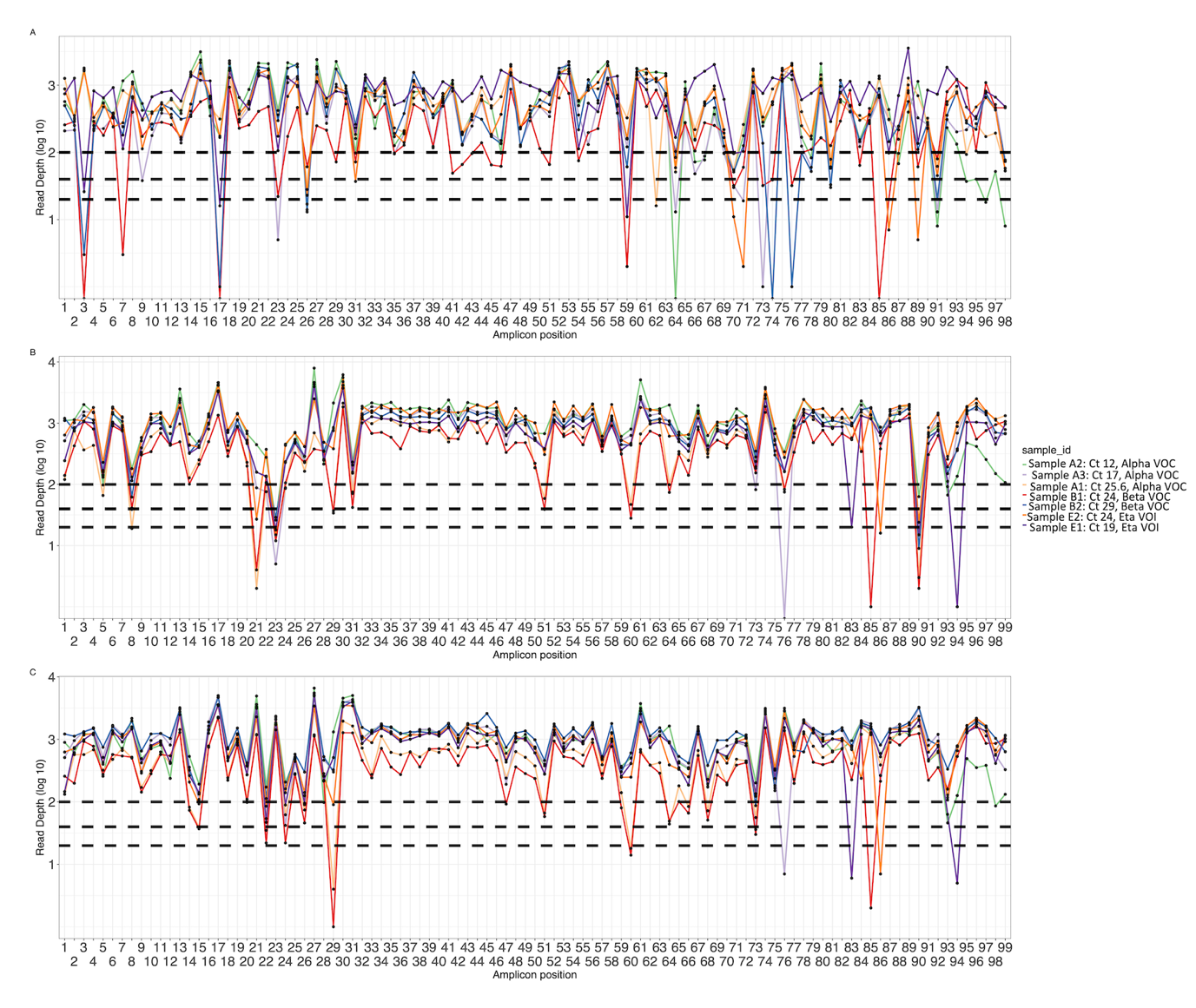


**Supplementary Figure 1:** Amplicon plots of seven sequences generated using (a) V3 primers, (b) V4 primers and (c) optimized V4 primers and classified as Alpha, Beta and Delta VOC. The curves show average depth in log scale (y axis) per amplicon (x axis). The horizontal dotted lines indicate amplicon depth cut-offs at 23, 50 and 100.


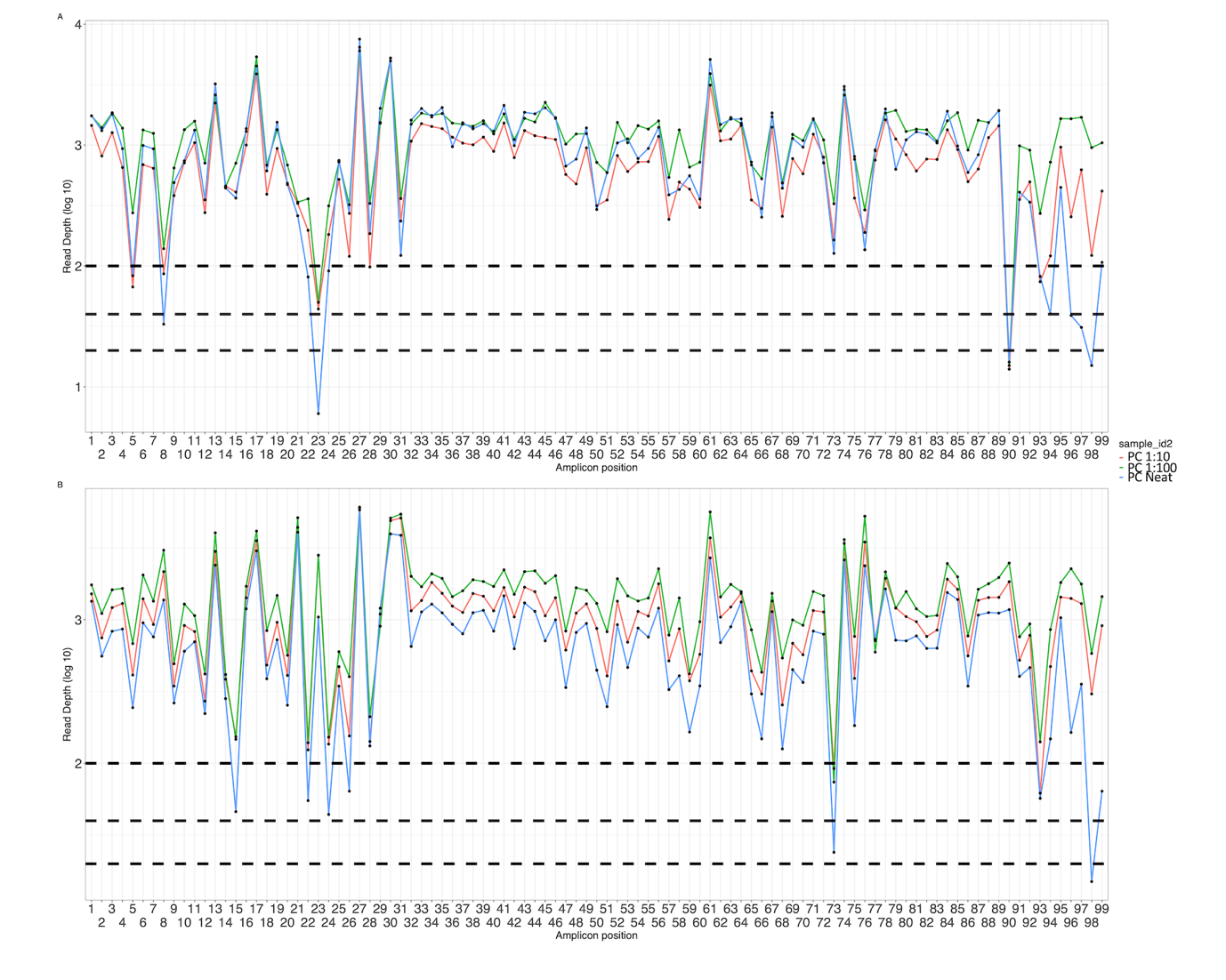


**Supplementary Figure 2:** Amplicon plots of serially diluted positive controls amplified using (a) V4 primers and (b) optimized V4 primers and classified as B.1. The curves show average depth in log scale (y axis) per amplicon (x axis). The horizontal dotted lines indicate amplicon depth cut-offs at 23, 50 and 100.

# Supplementary tables

**Supplementary Table 1:** Summary table of showing lineages among the samples used during optimization

| **Sample id** | **lineage** | **ambiguity_score** | **scorpio_call** | **scorpio_support** | **scorpio_conflict** | **version** | **pangolin_version** |
| --- | --- | --- | --- | --- | --- | --- | --- |
| sample_a1 | B.1.1.7 | 0.976849642 | Alpha (B.1.1.7-like) | 0.9565 | 0.0435 | PLEARN-v1.2.88 | 3.1.16 |
| sample_a2 | B.1.1.7 | 0.96723681 | Alpha (B.1.1.7-like) | 0.913 | 0.0435 | PLEARN-v1.2.88 | 3.1.16 |
| sample_a3 | B.1.1.7 | 0.895956734 | Alpha (B.1.1.7-like) | 0.8261 | 0.0435 | PLEARN-v1.2.88 | 3.1.16 |
| sample_b1 | B.1.351 | 0.948491538 | Beta (B.1.351-like) | 0.6429 | 0 | PLEARN-v1.2.88 | 3.1.16 |
| sample_b2 | B.1.351 | 0.94356826 | Beta (B.1.351-like) | 0.5714 | 0.1429 | PLEARN-v1.2.88 | 3.1.16 |
| sample_d1 | AY.16 | 0.870355731 | Delta (B.1.617.2-like) | 0.8462 | 0 | PLEARN-v1.2.88 | 3.1.16 |
| sample_d10 | AY.16 | 0.964492754 | Delta (B.1.617.2-like) | 0.8462 | 0 | PLEARN-v1.2.88 | 3.1.16 |
| sample_d11 | AY.46.4 | 0.934642147 | Delta (B.1.617.2-like) | 0.9231 | 0 | PLEARN-v1.2.88 | 3.1.16 |
| sample_d12 | AY.16 | 0.935171386 | Delta (B.1.617.2-like) | 0.9231 | 0 | PLEARN-v1.2.88 | 3.1.16 |
| sample_d13 | AY.16 | 0.931455633 | Delta (B.1.617.2-like) | 0.9231 | 0 | PLEARN-v1.2.88 | 3.1.16 |
| sample_d14 | AY.43 | 0.883011985 | Delta (B.1.617.2-like) | 0.9231 | 0 | PLEARN-v1.2.88 | 3.1.16 |
| sample_d15 | AY.46 | 0.880971026 | Delta (B.1.617.2-like) | 0.9231 | 0 | PLEARN-v1.2.88 | 3.1.16 |
| sample_d16 | AY.46.4 | 0.929855217 | Delta (B.1.617.2-like) | 0.9231 | 0 | PLEARN-v1.2.88 | 3.1.16 |
| sample_d17 | AY.46 | 0.916055626 | Delta (B.1.617.2-like) | 0.9231 | 0 | PLEARN-v1.2.88 | 3.1.16 |
| sample_d18 | AY.16 | 0.919062027 | Delta (B.1.617.2-like) | 0.9231 | 0 | PLEARN-v1.2.88 | 3.1.16 |
| sample_d19 | B.1.617.2 | 0.858018114 | Delta (B.1.617.2-like) | 0.8462 | 0 | PLEARN-v1.2.88 | 3.1.16 |
| sample_d2 | B.1.617.2 | 0.936228288 | Delta (B.1.617.2-like) | 0.9231 | 0 | PLEARN-v1.2.88 | 3.1.16 |
| sample_d3 | AY.16 | 0.931455633 | Delta (B.1.617.2-like) | 0.9231 | 0 | PLEARN-v1.2.88 | 3.1.16 |
| sample_d4 | AY.46 | 0.935435808 | Delta (B.1.617.2-like) | 0.9231 | 0 | PLEARN-v1.2.88 | 3.1.16 |
| sample_d5 | AY.46 | 0.925833125 | Delta (B.1.617.2-like) | 0.9231 | 0 | PLEARN-v1.2.88 | 3.1.16 |
| sample_d6 | AY.16 | 0.910823171 | Delta (B.1.617.2-like) | 0.7692 | 0 | PLEARN-v1.2.88 | 3.1.16 |
| sample_d7 | AY.46.4 | 0.867969369 | Delta (B.1.617.2-like) | 0.8462 | 0 | PLEARN-v1.2.88 | 3.1.16 |
| sample_d8 | AY.16 | 0.850362027 | Delta (B.1.617.2-like) | 0.7692 | 0 | PLEARN-v1.2.88 | 3.1.16 |
| sample_d9 | B.1.617.2 | 0.925025075 | Delta (B.1.617.2-like) | 0.9231 | 0 | PLEARN-v1.2.88 | 3.1.16 |
| sample_e1 | B.1.525 | 0.955154765 | Eta (B.1.525-like) | 0.9375 | 0 | PLEARN-v1.2.88 | 3.1.16 |
| sample_e2 | B.1.525 | 0.985085227 | Eta (B.1.525-like) | 1 | 0 | PLEARN-v1.2.88 | 3.1.16 |
| sample_nc1 | None |  |  |  |  | PANGO-v1.2.88 | 3.1.16 |
| sample_nc10 | None |  |  |  |  | PANGO-v1.2.88 | 3.1.16 |
| sample_nc2 | None |  |  |  |  | PANGO-v1.2.88 | 3.1.16 |
| sample_nc3 | None |  |  |  |  | PANGO-v1.2.88 | 3.1.16 |
| sample_nc4 | None |  |  |  |  | PANGO-v1.2.88 | 3.1.16 |
| sample_nc5 | None |  |  |  |  | PANGO-v1.2.88 | 3.1.16 |
| sample_nc6 | None |  |  |  |  | PANGO-v1.2.88 | 3.1.16 |
| sample_nc7 | None |  |  |  |  | PANGO-v1.2.88 | 3.1.16 |
| sample_nc8 | None |  |  |  |  | PANGO-v1.2.88 | 3.1.16 |
| sample_nc9 | None |  |  |  |  | PANGO-v1.2.88 | 3.1.16 |
| sample_nv1 | B.1.177 | 0.999766682 |  |  |  | PLEARN-v1.2.88 | 3.1.16 |
| sample_nv2 | B.1.530 | 0.984123223 |  |  |  | PLEARN-v1.2.88 | 3.1.16 |
| sample_nv4 | B.1 | 0.92637115 |  |  |  | PLEARN-v1.2.88 | 3.1.16 |
| sample_nv5 | B.1 | 0.835370823 |  |  |  | PLEARN-v1.2.88 | 3.1.16 |
| sample_nv6 | B.1 | 0.740969163 |  |  |  | PLEARN-v1.2.88 | 3.1.16 |
| sample_nv7 | B.1.36.35 | 0.653580402 |  |  |  | PLEARN-v1.2.88 | 3.1.16 |
| sample_nv8 | B.1 | 0.958707797 |  |  |  | PLEARN-v1.2.88 | 3.1.16 |

**Supplementary Table 2:** A table showing a summary of genome recovery when using ARTIC V3 compared to optimized V4 for 43 samples used in the optimization process

| Sample_id | Lineage | Genome Coverage (%) ARTIC V3 | Genome coverage (%) Optimized V4 | Differences in Genome coverage | Ct value |
| --- | --- | --- | --- | --- | --- |
| sample_d8 | AY.16 | 93.8 | 96.9 | 3.1 | 18.02 |
| sample_d1 | AY.16 | 96.2 | 99.6 | 3.4 | 16.71 |
| sample_d10 | AY.16 | 94.8 | 98.8 | 4.0 | 24.51 |
| sample_d12 | AY.16 | 94.8 | 99.6 | 4.7 | 22.43 |
| sample_d13 | AY.16 | 94.6 | 99.6 | 5.0 | 21.41 |
| sample_d3 | AY.16 | 94.1 | 99.6 | 5.5 | 22.15 |
| sample_d18 | AY.16 | 89.9 | 99.6 | 9.7 | 24.13 |
| sample_d6 | AY.46 | 94.8 | 97.8 | 3.0 | 24.6 |
| sample_d14 | AY.46 | 93.8 | 98.8 | 5.0 | 18.59 |
| sample_d15 | AY.46 | 90.2 | 95.6 | 5.4 | 27.76 |
| sample_d5 | AY.46 | 92.3 | 99.6 | 7.2 | 18.01 |
| sample_d7 | AY.46.4 | 94.9 | 98.0 | 3.0 | 25.42 |
| sample_d11 | AY.46.4 | 94.3 | 98.8 | 4.6 | 20.24 |
| sample_d4 | AY.46.4 | 88.6 | 98.8 | 10.2 | 24.23 |
| sample_nv6 | B.1 | 96.0 | 98.7 | 2.7 | 21.05 |
| sample_nv5 | B.1 | 93.3 | 97.0 | 3.7 | 19.8 |
| sample_nv4 | B.1 | 81.5 | 88.5 | 7.0 | 16.32 |
| sample_nc2 | B.1 | 40.2 | 91.6 | 51.4 | 25.88 |
| sample_a1 | B.1.1.7 | 97.0 | 97.8 | 0.8 | 25.69 |
| sample_a2 | B.1.1.7 | 96.0 | 99.6 | 3.5 | 12.57 |
| sample_a3 | B.1.1.7 | 92.3 | 98.9 | 6.7 | 17.96 |
| sample_nv1 | B.1.160 | 28.4 | 82.7 | 54.3 | 17.86 |
| sample_nv9 | B.1.177 | 99.6 | 99.6 | 0.0 | 18.28 |
| sample_b2 | B.1.351 | 95.1 | 97.0 | 2.0 | 29.92 |
| sample_b1 | B.1.351 | 94.8 | 99.6 | 4.8 | 24.16 |
| sample_nv7 | B.1.369 | 85.8 | 92.2 | 6.4 | 19.95 |
| sample_e1 | B.1.525 | 97.7 | 98.3 | 0.6 | 19.22 |
| sample_e2 | B.1.525 | 96.0 | 98.7 | 2.6 | 20.2 |
| sample_nv8 | B.1.530 | 97.8 | 98.9 | 1.1 | 29.43 |
| sample_nc1 | B.1.530 | 42.0 | 98.0 | 56.1 | 25.94 |
| sample_nv2 | B.1.617.2 | 74.3 | 93.1 | 18.9 | 17.45 |
| sample_d9 | B.1.617.2 | 94.9 | 98.7 | 3.7 | 19.72 |
| sample_d2 | B.1.617.2 | 93.3 | 98.4 | 5.1 | 19.36 |
| sample_d16 | B.1.617.2 | 91.5 | 99.6 | 8.0 | 21.38 |
| sample_d17 | B.1.617.2 | 88.7 | 97.8 | 9.1 | 27.96 |
| sample_d19 | B.1.617.2 | 87.7 | 98.8 | 11.2 | 22.89 |
| sample_nc3 | None | 56.0 | 73.9 | 18.0 | 26.47 |
| sample_nc5 | None | 27.7 | 66.9 | 39.2 | 22.62 |
| sample_nc6 | None | 25.2 | 67.3 | 42.1 | 20.98 |
| sample_nc7 | None | 14.7 | 62.4 | 47.7 | 29.73 |
| sample_nc8 | None | 19.5 | 69.4 | 49.9 | 24.71 |
| sample_nc9 | None | 18.0 | 68.1 | 50.1 | 18.11 |
| sample_nc10 | None | 7.8 | 68.1 | 60.3 | 26.08 |
